# A Single-Session Process-Based Cognitive-Behavioral Intervention Combined with Multimodal Rehabilitation Treatment for Chronic Pain Associated with Emotional Disorders

**DOI:** 10.3390/bs14040327

**Published:** 2024-04-15

**Authors:** Cristiana-Manuela Cojocaru, Cosmin Octavian Popa, Alina Schenk, Zsolt Jakab, Bogdan Andrei Suciu, Peter Olah, Horațiu Popoviciu, Simona Szasz

**Affiliations:** 1 The Doctoral School of George Emil Palade University of Medicine, Pharmacy, Science and Technology, 540142 Targu-Mures, Romania; cristiana-manuela.cojocaru@umfst.ro (C.-M.C.); alina.muresan@umfst.ro (A.S.); 2 Department of Ethics and Social Sciences, George Emil Palade University of Medicine, Pharmacy, Science and Technology, 540142 Targu-Mures, Romania; 3 Department of Counseling, Career Guidance and Informing Students, George Emil Palade University of Medicine, Pharmacy, Science and Technology, 540142 Targu-Mures, Romania; zsolt.jakab@umfst.ro; 4 Department of Anatomy and Morphological Sciences, George Emil Palade University of Medicine, Pharmacy, Science, and Technology, 540142 Targu-Mures, Romania; suciubogdanandrei@yahoo.com; 5 Department of Medical Informatics and Biostatistics, George Emil Palade University of Medicine, Pharmacy, Science and Technology, 540142 Targu-Mures, Romania; peter.olah@umfst.ro; 6 Department of Rheumatology, Physical and Rehabilitation Medicine, George Emil Palade University of Medicine, Pharmacy, Science and Technology, 540142 Targu-Mures, Romania; horatiu.popoviciu@umfst.ro (H.P.); szasz_fc@yahoo.com (S.S.)

**Keywords:** rheumatic conditions, chronic pain, anxiety, depression, psychological intervention, process-based cognitive-behavioral therapy, single-session intervention, psychosomatic medicine

## Abstract

Background and Objectives: Defined by chronic pain, rheumatic diseases are often co-occurring with anxiety and depression. Among the available psychological interventions, cognitive-behavioral therapies have an already-proven efficiency in these cases. However, the need to adjust their structure became ubiquitous during the post-pandemic period. Hence, the objective of this study was to investigate the impact of a single-session, process-based cognitive-behavioral intervention for patients with rheumatic conditions within an in-patient setting. Materials and Methods: A total of 31 participants (mean age 58.9 years) completed the single-session intervention. Assessments were conducted prior to the intervention, post-intervention and after one month. Results: Pearson’s correlations, paired samples T tests and a covariance analysis based on the Linear Mixed Model were performed for exploring the relations between baseline variables and evaluating the impact of the SSI intervention. Immediately after the intervention, a significant reduction in cognitive fusion (*p* = 0.001, *d* = 1.78), experiential avoidance (*p* = 0.001, *d* = 1.4) and dysfunctional behavioral processes was observed. At the one-month evaluation, participants reported decreased pain (*p* = 0.001, *d* = 1.11), anxiety (*p* = 0.004, *d* = 0.55) and depression (*p* = 0.001, *d* = 0.72). Conclusions: The single-session, process-based approach represents a promising intervention in healthcare contexts, as an integrative part of a multimodal rehabilitation treatment in patients with rheumatic conditions.

## 1. Introduction

Rheumatic conditions are highly associated with a poor quality of life, given their increased comorbidity with emotional disorders like anxiety and/or depression, which occur in approximatively 20% of patients or more [1,2,3]. Common emotional symptoms accompanying rheumatic diseases include anhedonia, withdrawal from activities, social isolation, sleeping problems and intense fear related to oneself and the future. This clinical picture has repeatedly been linked to the existence of dysfunctional thinking patterns regarding illness and pain, as well as unhealthy ways of reacting to these thoughts [4,5,6]. Specifically, the presence of chronic pain is considered one of the main mechanisms contributing to the onset of psychiatric disorders in association with rheumatic conditions [7,8]. These difficulties have a complex etiology, involving interactions between somatic, psychological and social elements [1,4]. Aside from the biological predisposition, other risk factors for the onset of this are aversive life events, such as interpersonal problems, grief, psychological abuse, financial difficulties and health-related concerns [9,10]. Particularly, due to the inherent unpredictability and burden, the COVID-19 pandemic was correlated with increased psychological distress and healthcare challenges in patients with rheumatic conditions, requiring the adjustment of therapeutic protocols and methods [11]. Along with the exacerbation of physical impairment, this context also affected psychological wellbeing and interpersonal relationships, correlating with enduring emotional symptoms and social isolation in this population [12] Also, the tendency to engage in unhealthy coping strategies was observed in association with the COVID-19 pandemic, including poor medication adherence and withdrawal from physical activity, which further emphasized the role of targeted interventions for promoting positive lifestyle changes [12,13,14].

The psychological theory of affective disorders was described as the “negative affect syndrome”, defined as the presence of persistent negative emotions in some individuals, characterized by multiple common features of anxiety and depression, as well as the high comorbidity rate between these disorders [15,16]. A main factor at the core of the negative affect syndrome is thought to be neuroticism, representing a personality trait associated with heightened stress reactivity and proneness to experience distressing emotions like anxiety and depression [17,18,19]. Moreover, the bidirectional relationship between emotional and physical symptoms generates a network defined by multimorbidity, which involves various functional difficulties [20]. Therefore, a multidisciplinary and integrative approach is recommended for the management of common rheumatic conditions, especially when emotional disorders are also present [21]. The first-line treatment involves the medical management, including disease-modifying antirheumatic drugs (DMARDs), glucocorticoids and topical nonsteroidal anti-inflammatory drugs, as well as psychiatric treatment like anxiolytics and antidepressants [22,23,24]. 

In addition to medical treatment, psychological interventions are highly recommended in these cases. In this regard, the efficiency of cognitive-behavioral therapies (CBT) is widely recognized in the management of chronic pain and associated emotional difficulties [25,26,27]. This approach outlines the role of pain-related thoughts in the occurrence of psychological distress, aiming to modify dysfunctional thoughts and correct specific cognitive distortions by promoting a healthier and realistic thinking style [28,29]. 

Symptoms within the anxious-depressive comorbidity, along with other disorders from the psychopathological spectrum, proved to be interrelated, sharing common roots and dysfunctional processes [30]. Starting from this premise, transdiagnostic psychological treatments led to the emergence of a new model, which describes psychopathological mechanisms as a network in which each process potentiates the others [31]. Confirming the association between the anxious and depressive clusters of symptoms, the CBT intervention for a specific anxiety disorder was correlated with improvements of other comorbidities, including depression [19,32]. In this way, process-based cognitive-behavioral therapy (PB-CBT) can be considered a new CBT branch that aims to change dysfunctional processes related to psychopathology with their functional counterparts, relying on the assumption that these mechanisms are modifiable and dynamic characteristics [33]. The PB-CBT is grounded in evolutionary theory, aiming to facilitate adaptation to various life contexts at the level of variation, selection and retention of strategies that operate at multiple dimensions of psychological functioning, including affectivity, cognition, attention, motivation and behavior [34].

Psychological interventions adapted for chronic pain were delivered in a variety of formats, including face-to-face, online, individual and group programs, with a typical duration of between 4 and 12 sessions [35]. However, the integration of medical and complementary treatments, along with the multiple medical appointments and time pressure inherent to the clinical environment, are associated with high withdrawal from psychotherapy in patients with chronic pain, which points to an imperious need to adjust these interventions to enhance benefits, while reducing drop-out rates [36,37]. So far, PB-CBT proved to be efficacious for alleviating anxiety and depression levels among both the general and chronic pain population, with very good acceptability [38,39,40].

This approach targets psychological inflexibility as a common process involved in the onset of psychological disorders associated with chronic pain, including cognitive fusion, experiential and behavioral avoidance [41,42,43]. Psychological inflexibility is defined as the excessive influence of thoughts and emotions over behavioral choices, resulting in the difficulty to adapt to different life contexts, including chronic pain [42,44]. Conversely, psychological flexibility refers to the ability to act concordant with personal values, despite experiencing uncomfortable emotions or physical sensations [45]. One of the main psychological inflexibility dimensions is cognitive fusion, which means that thoughts are appraised as general rules of functioning and not merely a perception of reality, so that the person takes decisions and acts according to the contents of their dysfunctional thoughts [42,46]. In chronic pain, this process is typically related to the evaluation of chronic pain as a global and pervasive experience (e.g., “I will always feel pain, meaning that my entire life is broken”). In other words, cognitive fusion occurs when patients tend to identify with the content of these negative thoughts, labelling themselves as “sick” and “worthless”, which in turn triggers negative emotions and social isolation [47,48,49]. In contrast, cognitive defusion helps separating thoughts from facts and enables a more detached perspective on them, decreasing their potential to generate distress [50]. At the level of emotions, experiential avoidance is characterized as the patient’s tendency to withdraw from distressing internal experiences, despite physical and psychological costs [42,51]. The healthier variant is represented by acceptance, or the willingness to fully experience emotions or sensations, whether pleasant or not, without attempts to control them, facilitating adaptation in the case of chronic pain [52,53]. From a behavioral point of view, difficulties are linked to the use of unhealthy coping strategies, inconsistent with the patient’s aspirations, especially in the context of a chronic health condition [40,43]. On the other hand, increasing values-based action was associated with less disability and elevated motivation in relation to chronic pain [54].

Against this background, as an alternative to the CBT short or medium-term intervention protocols, Single-Session Interventions (SSI) represent condensed treatments that can be considered useful ways to achieve the optimal results in a way that maximizes time resources [55,56]. These approaches consist of a unique psychotherapeutic session resembling the structure and contents of a typical psychological intervention that requires multiple meetings [55]. In chronic health conditions, the application of SSI with an average duration of 5.5 h, using a transdiagnostic, process-based framework was associated with moderate improvements related to functional measures, anxiety, depression and psychological inflexibility processes [57]. In this way, the main purpose of SSI is to provide solutions to specific challenges, in this case aiming to decrease psychological distress associated to chronic pain, by operating with similar concepts and techniques as full-length interventions [56,57]. For example, by comparing an SSI lasting for 2 h with a standard 8-session CBT protocol, Darnall et al. (2021) showed that emotional symptoms, pain-related distress and catastrophizing equivalently reduced among the treatment arms in patients with chronic low back pain [58]. Moreover, many patients with chronic medical conditions encounter various constraints that affect their adherence to standard psychological treatments, such as living remotely from medical institutions and hospitals which are typically located in more central places [59]. Hence the implementation of SSI is also motivated by practical considerations, enabling the delivery of psychological interventions along with the first-line medical treatment, corresponding to the requirements of institutionalized, in-patient settings. Table 1 depicts a comparative description including the main characteristics of SSI protocols and standard CBT interventions between 4 and 12 sessions. 

The PB-CBT model focuses on the identification and treatment of dysfunctional processes present at the cognitive, emotional and behavioral levels [60]. This approach allows a personalized intervention depending on the particularities of the patient’s psychopathological episode, which appears to be advantageous when compared to standard CBT interventions [61]. In addition, when delivered in the format of a single session, this type of intervention could serve as a suitable alternative for overcoming the obstacles to completing a standard CBT treatment, including time barriers and problematic treatment accessibility, especially for older adults with chronic pain [58]. Furthermore, the use of a PB-CBT approach facilitates the integration of multiple processes at the same time, including cognitive fusion, experiential avoidance and dysfunctional behaviors [57]. These advantages are particularly relevant during the period following the COVID-19 pandemic, enabling the use of various CBT techniques for improving psychological adjustment and overall quality of life [62,63].

Although these results are promising, further studies to investigate the benefits and feasibility of such brief interventions in clinical contexts are necessary. Based on the previous research literature, the main objective of the present study was to analyze the effects of an in-patient process-based SSI for patients with rheumatic diseases presenting chronic pain. First, starting from the PB-CBT model, we expected to find correlations between baseline measures of pain, fatigue, anxiety and depression, on the one hand, and the psychological inflexibility processes of cognitive fusion, experiential avoidance and behavioral strategies, on the other hand. Second, we expected that cognitive fusion, experiential avoidance and dysfunctional behavioral processes would reduce after the SSI, while the functional coping strategies would improve for those patients. Third, another hypothesis was that subjective pain, fatigue, anxiety and depression would decrease after the multimodal rehabilitation treatment, including an SSI based on PB-CBT. 

## 2. Materials and Methods

Participants. The sample size was calculated using the G-Power software version 3.1 [64] for a moderate effect size set at 0.5, an expected power of 0.8 and an alpha error probability of 0.05. A total number of 31 participants (aged M = 58.9, SD = 12.03) who presented a musculoskeletal condition diagnosed by a rheumatologist and were hospitalized for at least 1 week within the Rheumatology section of the Targu-Mures County Emergency Clinical Hospital participated in this study. Further inclusion criteria were: (1) age over or equal to 18 years old; (2) the presence of a chronic pain condition for at least 3 months; (3) the fulfilment of psychodiagnosis criteria for generalized anxiety disorder and/or major depressive disorder, according to the Structured Clinical Interview for the DSM-5—Clinician Version. Exclusion criteria were: (1) the existence of severe cognitive impairment; (2) the presence of severe psychiatric disorders; (3) not being able to properly understand the Romanian language. 

Ethical considerations. This study was approved by the Institutional Ethics Committee of the Targu-Mures County Emergency Clinical Hospital from 27 July 2023 under the number 19,622. Before enrolling in the study, participants were informed regarding the objectives and procedures of the research and signed the informed consent.

Measures

The DECAS Personality Inventory [65] was developed and validated on the Romanian population, based on the Five Factor Model of Personality [66], providing accurate estimates of important personality dimensions in relation to clinically relevant variables such as emotional distress. Particularly, the Emotional Stability subscale measures neuroticism, which is defined as the tendency to experience negative emotions more easily, encounter difficulties when confronted with stressful situations and rely on unhealthy coping strategies. At higher levels, there is a high overlap between this personality dimension and the occurrence of emotional disorders [67]. The subscale includes 19 items and asks responders to decide whether a specific affirmation is true or false in their case most of the time. The internal consistency demonstrated in the original study was good, as reflected by the obtained Cronbach’s alpha coefficient of 0.75 [68]. 

The Structured Clinical Interview for the DSM-5—Clinician Version (SCID-5-CV) [69] refers to a semi-structured clinician-rated tool based on the diagnosis criteria listed in the Diagnostic and Statistical Manual of Mental Disorders, Fifth Edition (DSM-5) [70]. Using a series of guiding questions, the interviewer decides whether key psychopathological symptoms are present or not in the case of a particular person, the instrument proving good discriminative properties. Specifically, for anxiety disorders, sensitivity and specificity were estimated at 0.94 and 0.78, respectively. At the same time, for depressive disorders, both sensitivity and specificity were 0.84. Also, the SCID-5-CV demonstrated moderate, albeit reasonable inter-rater agreement rates, with Kappa coefficients of 0.34 for anxiety disorders and 0.69 for depressive disorders [71]. However, other investigations estimated a Kappa coefficient of 0.73, along with a positive agreement rate of 73% for any anxiety disorder [72]. Therefore, it was concluded that the interview can reliably be used for screening purposes in both in-patient and out-patient settings. 

The Visual Analogue Scale (VAS) is a single-item measure applied in the assessment of both physical and psychological variables [73]. Given their practical utility and high feasibility, such scales were traditionally implemented for evaluating the subjective pain intensity in various medical contexts. Typically, the VAS requires respondents to rate the severity of the symptom on a 10 cm scale, from 0 = not present to 10 = worst imaginable intensity of the symptom [74]. Also, fatigue ratings were evaluated with the VAS in patients with rheumatoid arthritis, indicating similar properties to alternative fatigue measures in terms of sensitivity and association to clinical outcomes [75]. In addition, this approach was used for evaluating psychological dimensions, like depressive mood and showing proper convergent and divergent validity [76,77]. Indeed, the VAS for depression proved a correlation of 0.61 with the total score of other validated and widely employed measures of depression, like the Patient Health Questionnaire-9 (PHQ-9) [76]. Finally, the same approach tested for anxiety led to equivalent results in terms of validity, as indicated by the correlations between VAS and other renowned tools, such as the Hamilton Rating Scale for Anxiety (r = 0.60), or the Hospital Anxiety and Depression Scale—Anxiety subscale (r = 0.74) [77]. 

The Process-Based Assessment Tool (PBAT) [78] uses a behavioral approach to evaluate processes of change, which are highly cited, especially in the context of PB-CBT [34]. Unlike most evaluation scales, this instrument is defined as an “item pool”, integrating 18 individual items that depict positive and negative behavioral responses. In this way, there are two items corresponding to the evolutionary dimension of variation, namely the ability to change one’s behavior when it is beneficial (positive; item 1) and, the opposite, an incapacity to change and being stuck (negative; item 12). Retention is also reflected in two items: indicating one’s struggle to continue engaging in important action (positive; item 4) along with remaining stuck to previous strategies that are currently inefficient (negative; item 10). Finally, most contents are related to the evolutionary dimension of selection. On the one hand, positive selection mechanisms are presented in 8 items, as follows: the ability to experience a wide range of emotions (affective component; item 3), the choice of actions that may improve one’s physical health (item 6), paying attention to important goals and actions (motivation component; item 8), finding personally relevant ways for challenging oneself (need for competence; item 11), using one’s thinking to improve life (cognitive component; item 13), attempts to connect with meaningful moments (attention component; item 14) and significant people (social connection; item 15), along with choosing to do things that are personally important (item 16). On the other hand, negative selection mechanisms are included in the next 5 items: doing actions that hinder one’s connection to other people (social connection; item 2), failing to find a meaningful way to challenge oneself (need for competence; item 5), thinking in an inefficient way (cognitive component; item 7), performing various actions as a result of social desirability (motivation component; item 9), engaging in actions that are detrimental to one’s health (item 17), as well as facing difficulties in expressing emotions (affective component; item 18). Items are typically rated using a digital-analogue scale from 0 = strongly disagree to 100 = strongly agree. However, due to the setting of this study, we applied a classical pen-and-paper approach, using the same ordinal range. The original research on PBAT criterion validity showed that positive items predict good health and vitality, whereas negative processes are linked to sadness, anxiety, stress, anger and lack of social support [78]. 

The Acceptance and Action Questionnaire (AAQ-II) [79] refers to a self-report 7-item scale for measuring experiential avoidance, defined as the tendency to ward off difficult internal experiences, especially negative emotions (e.g., “I worry about not being able to control my worries and feelings” or “Emotions cause problems in my life”). Responses are rated using a 7-point Likert scale (starting from 1 = never true to 7 = always true). Total scores represent the sum of individual items, higher values indicating heightened experiential avoidance, with a higher possible score of 49. The mean Cronbach’s alpha coefficient obtained in the original validation study was 0.84, which suggests a very good reliability, along with the criterion and predictive validity established through comparisons with other symptom-oriented scales for assessing emotional disorders [79]. The Romanian validation of the scale resembled these findings, with a reliability coefficient of 0.80 [80]. In the present study, the Cronbach’s alpha coefficient values ranged from 0.65 to 0.76. 

The Cognitive Fusion Questionnaire (CFQ) [81] is a 7-item scale that measures the tendency to consider thoughts as general rules and rely on them when making behavioral decisions, which amplifies the individual’s adjustment difficulties. As reflected in the content of the questionnaire (e.g., “I get so caught up in my thoughts that I am unable to do the things that I most want to do” or “It’s such a struggle to let go of upsetting thoughts even when I know that letting go would be helpful”), this mechanism is effortful, limiting personal coping resources. A 7-point Likert scale (starting from 1 = never true to 7 = always true) is employed for rating, adding up to a maximum final score of 49. From the perspective of psychometric properties, the CFQ demonstrated very good internal consistency, with Cronbach’s alpha coefficients ranging from 0.88 to 0.93 in the original validation study, depending on the participants’ characteristics. Also, the scale has proper criterion validity, as shown by the correlations with other instruments measuring psychological processes and clinical manifestations of various psychopathologies [81]. By using the Romanian translation of CFQ, we obtained similar reliability, with Cronbach’s alpha levels of 0.83 and 0.84 at pre- and post-test measures, respectively. 

**Figure 1:**
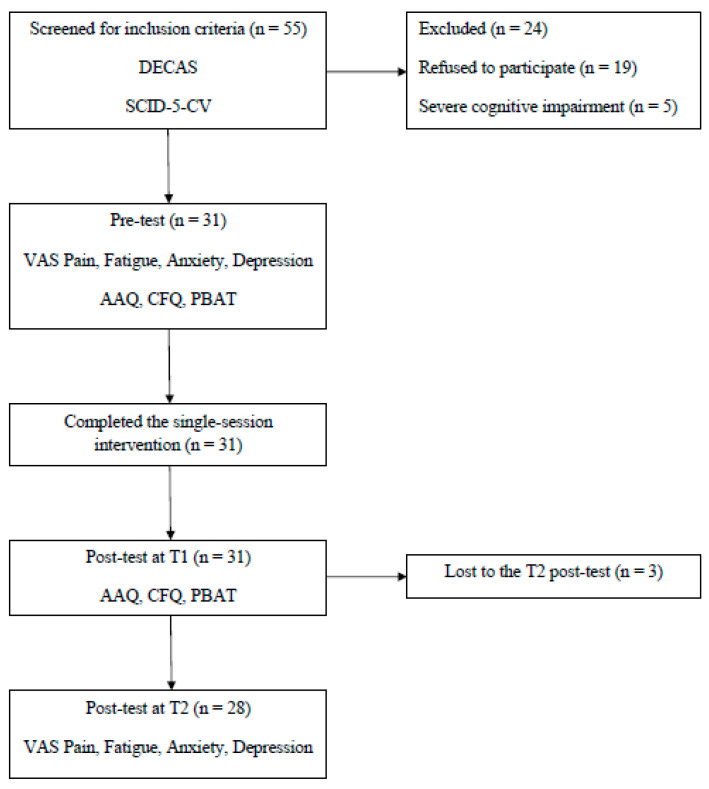
The flow diagram of the study procedure.

Design and procedure. The present study is an uncontrolled trial, comprising an intervention group with no control arm. The participant flow is depicted in Figure 1. A quasi-experimental design was used, all participants fulfilling the inclusion criteria being further involved in the intervention. A convenience sampling was applied, relying on the patients’ agreement and willingness to participate in this research. The screening involved the DECAS Personality Inventory. More specifically, the composite T scores for the Emotional Stability (Neuroticism) subscale were extracted for each participant by using the DECAS online platform [65]. Participants that scored lower than the normative level based on their age and gender in Emotional Stability were considered for further screening. This initial assessment was doubled by the application of the SCID-5-CV—generalized anxiety disorder and major depressive disorder sections. Participants who met the criteria for one or both diagnoses were included in the study. A total number of 31 participants were enrolled and completed the SSI, based on established techniques and protocols of PB-CBT for chronic pain [82,83,84]. The intervention was carried out at the bedside, in an individual format, within two days after the hospital admission and lasted between 70 and 80 min. So far, SSI interventions had a highly variable duration between 40–50 min [85] and 8 h [57,58]. However, many of these programs were delivered in a workshop format, in different settings and enrolled participants with diverse diagnoses. Thus, the SSI applied in our study considered the optimal length for an individual session within the in-patient context, where participants underwent a multimodal treatment involving medical and physical therapy as first-line treatments, as well as the potential constraints related to the attention span and concentration ability which characterize chronic pain conditions [86]. The SSI consisted in four major components, each lasting for about 15–20 min. The first part involved a psychoeducation about the tendency to engage in experiential avoidance as a response to pain, withdrawing from many meaningful activities, or preventing the experience of difficult sensations or emotions. The ways in which avoidance could occur in daily living was discussed with patients, who were asked to think about personal examples. The second step used a mindfulness technique, providing a healthier way for patients to cope with unpleasant feelings. After a brief description of the body scan approach, the exercise was performed, requiring participants to close their eyes, start to focus on their breath and then on each bodily part, only noticing sensations, without evaluating them in any way. The third part introduced cognitive fusion as a dysfunctional way of dealing with negative thoughts that increases distress. Cognitive defusion was explained with the help of metaphors (e.g., thoughts could be seen as clouds in the sky or people passing by the street). The practical exercise used a guided imagery script in which patients were encouraged to visualize a landscape of streaming water, along with their thoughts on floating leaves, just observing them from the edge. The fourth component was related to the concept of personal values and applied a simple comparison between significant life areas and the energy sources of a recharging battery to help participants understand the role of meaningful action in motivation and subject wellbeing. The session ended with an action plan that integrated up to five behavioral goals for the upcoming month, which were established in a collaborative way with patients, according to the previously identified life values. The steps of the single-session protocol are presented in Table 2. The pre-test involved the administration of a brief demographic questionnaire, the VAS scales for pain, fatigue, anxiety and depression, as well as the evaluation of psychological flexibility processes with the AAQ, CFQ and PBAT, respectively. The post-test at T1 was conducted after 3–5 days following the intervention, including the use of AAQ and CFQ scales. The post-test at T2 was conducted telephonically at one month after the hospital admission, asking participants to evaluate the intensities of pain, fatigue, anxiety and depression during the previous two weeks using the VAS scales. The selection of variables for the T1 and T2 assessment relies on the research indicating the classification of experiential avoidance, cognitive fusion and behavioral variables as dynamic states, while highlighting the relative stability of physical and emotional functioning components [87,88,89]. Also, the instruments implemented in the present study allowed for the evaluation of T1 variables immediately after the intervention, while the T2 measures required a longer time span between the prior and post-assessment. The contextual discrepancy between T1 and T2 was another aspect contributing to the selection of the VAS scales only for the one-month telephonic administration. Based on past studies showing a strong correlation between psychological inflexibility and functional variables, we assumed a pattern of interdependent relationships between cognitive fusion, experiential avoidance, dysfunctional behaviors, perceived pain and emotional disorders, especially given the post-pandemic context [90].

Statistical analyses. Outcomes of the present study were analyzed using the IBM SPSS software, version 29.0.2. The statistical analysis involved the means and standard deviations for computed variables, based on the total scores for AAQ and CFQ and the raw scores for the VAS measures and each PBAT item. Skewness and kurtosis values were used as indicators of data normality. For both skewness and kurtosis, the ±2 threshold was assumed for indicating that data are normally distributed [91]. Also, data linearity was explored through the visual inspection of Q-Q plots. Since the underlying assumptions of normality, linearity, data continuity and absence of significant outliers have been met, Pearson’s correlations and paired samples *T* tests, along with the Mixed Linear Model were selected for statistical analysis. As a first step, Pearson’s correlations were performed with the purposes of investigating and establishing the relations between different sets of variables at baseline, including subjectively evaluated health and mood components (pain, fatigue, anxiety and depression), as well as psychological inflexibility and behavioral processes. As a second step, paired-sample *T* tests were carried out, in order to analyze the intervention effects, by comparing the difference in the mean level of experiential avoidance and cognitive fusion from the pre-test to the T1 post-test, along with the impact on the subjective ratings of pain, fatigue, anxiety and depression from pre-test to the T2 post-test. The data imputation method was applied to estimate missing values, by calculating the average scores for pain, fatigue, anxiety and depression at the T2 assessment and using these values for replacing the lost data in the analysis. Effect sizes were estimated by using the Cohen’s d, as well as the Hedges’ g as a correction for multiple comparisons. As a third step, a Linear Mixed Model analysis was conducted to investigate potential socio-demographic covariates influencing the difference in scores between pre- and post-tests. For this analysis, data were restructured using the following codes for the nominal variables: for gender, “1 = female”, “2 = male”; for marital status, “1 = married/in a committed relationship”, “2 = single/widowed”; for occupational status, “1 = employed”, “2 = unemployed/retired”; and for diagnosis, “1 = inflammatory pain/arthritis”, “2 = mechanical pain/arthrosis”. The model included the outcome measure as dependent variable, time as fixed factor, as well as the socio-demographic variables (i.e., age, gender, marital status, occupational status and diagnosis) as covariates, using the unstructured repeated covariance type.

## 3. Results

### 3.1. Descriptive Characteristics

Table 3 comprises the demographic particularities of our sample, along with the average baseline VAS scores regarding the major physical and mood components. The average participant was a female, around 59 years old, married, currently retired and graduated a form of secondary education (professional or high school). The intake/admission screening/assessment indicated an average extreme severity of subjectively reported pain and fatigue, along with moderate to high anxiety and depression ratings.

### 3.2. Baseline Measures

The results of the correlation analyses performed on baseline measures, including health components, psychological flexibility and behavioral processes, are depicted in Table 4. Pain scores were significantly correlated with subjective anxiety levels (r = 0.40), along with the processes of cognitive fusion (r = 0.43) and the PBAT item Hurt Connection (r = 0.40). Also, fatigue was moderately and positively associated to depression scores (r = 0.41), as well as several behavioral processes, including the PBAT items Important Challenges (r = 0.49), Connect to People and Personal Importance (r = 0.41), while negatively associated to PBAT item Stuck, unable to change (r = −0.37). In addition, anxiety was positively correlated with experiential avoidance (r = 0.37) and the PBAT item Thinking Got In the Way (r = 0.42). Regarding the relation between psychological flexibility and behavioral processes, the PBAT item Hurt Health was positively and strongly associated to cognitive fusion (r = 0.60) and experiential avoidance (r = 0.45), respectively. In contrast, negative correlations emerged between experiential avoidance and the PBAT items Thinking Helped Life (r = −0.41) and Struggle Connect Moments (r = −0.35). In a similar way, the PBAT item Experience Range Emotions was inversely correlated with both experiential avoidance (r = −0.39) and cognitive fusion (r = −0.36). As expected, we also found a strong positive association between experiential avoidance and cognitive fusion (r = 0.60). 

### 3.3. Intervention Outcomes

#### 3.3.1. Results at T1

The results are summarized in Table 5, showing significant change in psychological inflexibility dimensions immediately after the intervention. Both cognitive fusion (t = 9.96, *p* = 0.001) and experiential avoidance (t = 7.79, *p* = 0.001) were significantly reduced at post-test, as compared to baseline. In regard to the behavioral strategies, a statistically significant difference was observed in relation to five processes. First, participants showed an improved ability to change their behavior (PBAT item 1; t = −4.37, *p* = 0.001) and to engage in behaviors that helped physical health (PBAT item 6; t = −4.32, *p* = 0.001). Also, a reduced proneness to comply with other’s wishes without taking one’s own needs into account (PBAT item 9; t = 3.86, *p* = 0.001), along with the lower tendency to feel stuck to unproductive working strategies (PBAT item 10; t = 3.27, *p* = 0.003) and a decreased likelihood to behave in ways that would damage health (PBAT item 17; t = 4.05, *p* = 0.001) were observed after the psychological intervention. A qualitative evaluation of the magnitude of improvements observed for each psychological process from baseline to the T1 assessment is depicted in Table 6. 

#### 3.3.2. Results at T2

The outcomes of the assessment conducted one month after the intervention are synthesized in Table 7. Specifically, the T2 assessment indicated a statistically significant decrease in pain ratings (t = 6.21, *p* = 0.001). In addition, both anxiety (t = 3.08, *p* = 0.004) and depression (t = 3.96, *p* = 0.001) scores were lower at the T2 post-test, in contrast to the pre-test. However, we did not observe a statistically significant reduction in fatigue scores at the T2 post-test.

#### 3.3.3. Results of the Linear Mixed Model

The results of the repeated measures covariance analysis resembled the parameters indicating the intervention effects obtained using the paired samples T test. Table 8 includes the estimated coefficients for each outcome measure, along with the corresponding standard error. Among the covariates, only age reached the significance threshold for anxiety, with t = 2.86, *p* = 0.008, estimate 0.11 (0.03; 0.19), SE = 0.03, and PBAT item 9—Complying, with t = −2.54, *p* = 0.01, estimate −0.91 (−1.66; −0.17), SE = 0.36. 

## 4. Discussion

First, the correlational analysis indicated a pattern of associations between psychological inflexibility processes and reported physical or emotional symptoms at baseline in the expected direction. This finding supports the idea that several interdependent mechanisms could underpin the comorbidity between rheumatic diseases and emotional disorders [92]. Specifically, in our study, experiential avoidance and cognitive fusion were positively associated with the tendency to behave in ways that would damage one’s health. This could point to the existence of an indirect pathway explaining the link between psychological inflexibility and health-related problems through unhealthy behavioral choices [93]. Likewise, the negative associations between psychological inflexibility processes, the existence of a broad emotional repertoire, and the ability to change thoughts to improve the mood outlined the connection between beliefs, feelings and behaviors that ultimately marks the boundary between psychopathology and healthy psychological functioning [94,95]. Also, perceived pain intensity was positively correlated to the tendency to engage in behaviors that damaged meaningful interpersonal relationships, which is in line with previous studies showing a link between interpersonal processes and pain adjustment [96]. In addition, the experience of fatigue was associated with perceived lack of meaningful reinforcements and incapacity to change inefficient strategies, akin to past research showing a connection between perceived energy level and motivational features in chronic pain [97]. 

Second, our results showed that a brief and compacted intervention can promptly improve psychological flexibility in patients with rheumatic diseases and emotional comorbidities. This is in line with previous findings indicating that a single workshop based on PB-CBT can reduce psychological distress in patients with chronic pain [98]. In our study, participants proved to be less prone to engage in experiential avoidance and more willing to experience uncomfortable emotions and sensations immediately after the intervention. This is concordant to a large body of evidence that demonstrated the benefits of process-based therapies to increase acceptance, especially in relation to chronic pain [99,100,101]. Furthermore, the level of cognitive fusion decreased shortly after the intervention. In a similar light, it has been proven that a single-session treatment is effective for modifying common cognitive features associated with emotional distress in chronic pain patients [102,103,104]. 

Third, at the behavioral level, the general direction was related to factual changes that promoted health-related activities. As expected, during the hospitalization, participants had a higher probability to engage in activities supporting physical health. Along with the inclusion of the intervention component focused on values and committed action, involving a concrete action plan with specific objectives for facilitating a balanced lifestyle, we believe this behavioral change could be strongly correlated to the inpatient context, defined by a multimodal approach in the treatment of rheumatic conditions [105]. This aligns with previous studies concluding that patients’ motivation and treatment adherence are correlated with good-quality medical care, using an integrative framework for targeting multiple areas of functioning [106,107,108]. Similarly, several investigations proved that encouraging values-based living is linked to better functioning that includes personalized action plans which facilitate the attainment of meaningful goals, despite the presence of chronic pain and potential physical constraints [109,110,111,112]. Interestingly, the likelihood of complying with others’ wishes without considering one’s own needs was also reduced, which points to an increased autonomy in the face of long-lasting health challenges [111]. Indeed, previous research indicated that promoting patients’ self-determination is related to benefits in terms of disease-related behaviors in psychosomatic medicine [113,114,115,116,117]. 

The qualitative analysis of changes in psychological processes from baseline to the first evaluation after the intervention showed that half of participants showed at least mild positive changes in cognitive fusion and experiential avoidance. This aligns with previous studies indicating that cognitive and emotional processes are dynamic and modifiable characteristics, presenting beneficial results after a brief intervention [60,88]. Positive behavioral changes were also observed after the SSI. However, these outcomes involve narrower topics, thus the participants’ responses present more variability. This aspect could reinforce the possibility of developing tailored CBT interventions involving different lengths and distribution of protocol dimensions for addressing the individual needs of patients with rheumatic conditions and comorbid emotional disorders [61,100,105].

Moreover, one month after the intervention, participants reported improvements related to the symptom components. Namely, as anticipated following hospitalization, significant changes were observed regarding pain perception. In addition, we could assume that this outcome is also concordant with other research showing that process-based psychological treatments have a positive impact on pain intensity and associated disability [118,119,120,121]. Furthermore, subjective anxiety and depression levels decreased after the in-patient treatment, compared to the baseline assessment. Multiple investigations resembled these findings, proving that working on dysfunctional cognitive and emotional mechanisms may notably alleviate distress in patients with chronic pain [122,123,124,125,126]. This emphasizes that even a brief intervention can facilitate the development of core skills for improving the behavioral management of chronic pain in the long run, providing a condensed package of techniques for dealing with challenging emotions and sensations, like cognitive defusion, mindfulness and action planning. We believe this outcome could serve as a premise for longitudinal investigations that plan follow-up assessments at different intervals to indicate the maintenance of these benefits. Also, the SSI could be enhanced, including a set of strategies to increase patients’ empowerment and commitment with the assumed action plan, such as guided discovery or motivational interviewing methods [37].

Given the specificity of the intervention implemented in the present study, it is noteworthy to acknowledge the potential contribution of a multitude of factors to explaining our findings. Namely, several categories of pain-relieving medications, including serotonin-norepinephrine reuptake inhibitor (SNRI) antidepressants like duloxetine or milnacipran have a positive impact on both pain and emotional symptoms, particularly depression, improving functional outcomes in patients with chronic pain [127,128]. In the same light, physical therapies can significantly reduce pain-related distress, especially when applied along with educational interventions [129]. Furthermore, there is evidence that a good therapeutic alliance may strengthen the outcomes of various interventional techniques [130]. It is possible that this relational exchange occurring between practitioners and chronic pain patients functions as a placebo, facilitating the changes observed after the treatment [131]. In this way, along with the SSI implemented in our study, we believe that all these factors could generate a combined beneficial effect, leading to the symptomatic improvement and decrease in dysfunctional psychological processes that we noticed. In addition, when covariates were introduced within the investigation, we obtained a significant effect of age on anxiety and patients’ tendency to continue using dysfunctional coping strategies. Hence, patients may present differential acceptability and responsivity to the SSI content according to their age. This aspect could become the subject of upcoming studies applying SSI approaches, bringing insights for developing more tailored psychological interventions in chronic pain.

Nonetheless, several limitations could impede the interpretation of the results in the present study. There are methodological constraints that hinder the generalizability of our findings. First and foremost, since our study did not include a control group, the effects of the intervention could not be differentiated from other factors influencing the subjective experience of participants. While an uncontrolled design may provide the framework for more naturalistic research and resemble real-life circumstances, we acknowledge that a less rigorous trial generates a series of shortcomings regarding the validity and fidelity of results. Second, we grant that failing to include measures of cognitive fusion, experiential avoidance and behavioral processes, aside from the physical and emotional functioning variables at the one-month assessment, does not permit monitoring the maintenance of the observed positive changes related to psychological flexibility. Also, the connection between potential mechanisms of change and clinical symptoms could not be established in our study, which points to the need to continue the research on the effects of this protocol on chronic pain patients in the long run, with a closer look at the associations between key variables. In this way, an important limitation is the incapacity to rule out the possibility of confounds that could influence the final outcomes. For example, other components of the treatment, like administered medication, especially antidepressant medications used for the alleviation of chronic pain or physical therapy in the form of kinesiotherapy, physiotherapy or hydrotherapy sessions that participants attended during the hospitalization period, could involve benefits that extend beyond somatic components, improving the psychological adjustment in patients with rheumatic diseases. Also, the benefits observed after the hospitalization could be linked more to the context, especially the fact that participants returned home and spent more time with their families, which could create more opportunities for experiencing positive emotions and participating in different happy events, lifting the emotional burden of living with chronic pain. Third, our research included self-report measures, meaning that the results could be influenced by social desirability. This concern is particularly valid given the institutionalized environment, in which the psychological approach was integrated among various treatment components and the psychotherapist was perceived as part of the healthcare team. Specifically, participants’ responses could have been motivated by their need to be approved by the medical staff, particularly at the T1 evaluation conducted during the hospital stay. Hence, it is not possible to draw firm conclusions regarding the efficiency of the SSI implemented in the present study. While our results require a judicious and attentive interpretation, future work in this respect might consider incorporating more objective measures and different settings for the assessment and intervention phase. Fourth, another limitation assumed by the authors is related to the measurement of anxiety and depressive symptoms, rated with a single item instrument. Fifth, the sample size used in our study was small, especially considering the nature of a single-session approach implemented in the medical context, failing to provide a wide range of demographic and psychological characteristics. Sixth, we did not include longitudinal assessments for observing the evolution of these outcomes over time. Therefore, we believe future analyses could overcome these limitations by including larger sample sizes, passive and active control groups, along with additional instruments for assessing the intensity of clinical manifestations and functional aspects follow-up intervals. At the same time, further studies could include multiple assessment tools and methods for an extensive evaluation of both symptoms and psychological processes to monitor fluctuations from one time point to another, integrating clinician-rated self-reports, as well as more objective measures of health parameters.

Clinical implications. The outcomes of the present study may inform the development of targeted protocols aiming to reduce emotional distress in patients with chronic pain, as part of interdisciplinary treatment packages [24,132,133]. The utility of the SSI format lies in the ability to achieve meaningful improvements in a very short time, which transforms it into an efficient tool to match the challenges of delivering psychotherapeutic interventions within in-patient contexts [57,134]. Also, by encouraging health-related action, these interventions could successfully prevent the various side effects of medication misuse in patients with psychosomatic conditions [135,136]. In addition, such approaches can be easily integrated in the routine care of patients with rheumatic diseases, being easily applicable by medical professionals for increasing patients’ motivation and treatment compliance above and beyond the hospitalization period [137,138,139]. In this way, we believe that the adoption of these treatment practices could have a positive impact on the burden of multimorbidity for the healthcare system as a whole in the long run.

## 5. Conclusions

This study showed that a brief process-based psychological intervention was associated with immediate improvements related to the cognitive, emotional and behavioral functioning in patients with rheumatic diseases and comorbid emotional disorders. Also, this approach could positively impact the experience of pain, anxiety and depression, outside of the in-patient context. However, further studies are required for drawing firm conclusions regarding the benefits of single-session approaches using a process-based framework with the medical milieu. 

## Figures and Tables

**Table 1:** A comparative analysis between SSI CBT and standard CBT protocols involving main intervention particularities.

	SSI CBT	Standard CBT
**Advantages**	Increased feasibility for the healthcare/ in-patient setting; Improved accessibility; Compact and comprehensive intervention; The provision of all protocol steps to each beneficiary; Efficient use of resources; Larger number of beneficiaries in a shorter amount of time.	Detailed approach to each protocol step; Improved therapeutic rapport and continuity of the intervention; More time for results consolidation; Facilitation of follow-up and progress assessment.
**Potential Disadvantages**	Fewer time dedicated to each protocol step, including relapse prevention.	Lower compliance and motivation.
**Time** **(Average length)**	Between 40–50 min and 8 h	4–12 h
**Cost**	Low	High
**Limitations**	Difficulties related to longitudinal and follow-up assessments.	Accessibility barriers and time constraints.
**Attrition**	Low	High
**Required qualifications**	Formal certification in Cognitive-Behavioral Therapy	Formal certification in Cognitive-Behavioral Therapy

**Table 2:** The single-session protocol implemented in the study.

Topic	Description
**1. Dealing with pain**	The tendency to avoid difficult internal experiences, including pain, as well as the consequences of this strategy were discussed. The acceptance alternative was introduced as a healthier way to cope with pain.
**2. Present moment awareness**	Fewer time dedicated to each protocol step, including relapse prevention. This part was more practical, involving the use of a brief body scan mindfulness exercise. This practice focused on breathing and the acknowledgement of physical sensations. Also, the “expansion” technique was implemented, encouraging participants to create space for the pain, by delimitating it from other bodily parts and focusing their attention to various characteristics of this sensation.
**3. Practising cognitive defusion**	The idea that people could get entangled in their thoughts, especially when confronted with difficult experiences was outlined. The concept of cognitive defusion was then explained to participants through practical examples. Specifically, the “Leaves on a stream” technique was applied to promote an observer stance and facilitate a functional attitude towards pain-related thoughts.
**4. Towards a values-based life**	A discussion related to the role of personal values for developing a sense of meaning was initiated. For this purpose, a metaphor based on the “Batteries exercise” was presented, pointing to potential discrepancies between deeply held values and the behaviors in which participants may involve. Relying on this framework, a brief action plan based on the identified personal values focusing on the upcoming two weeks was developed for each participant.

**Table 3:** Descriptive characteristics of the study sample.

Age (M, SD)	58.9 (12.03)
Gender (N,%)	
Female	29 (94)
Male	2 (6)
Marital status	
Married	25 (81)
Single	1 (3)
Widowed	5 (16)
Occupational status	
Employed	4 (13)
Unemployed	8 (26)
Retired	19 (61)
Main diagnosis	
Ankylosing spondylitis	1 (3)
Chronic post-surgical pain	2 (7)
Coxarthrosis/Gonarthrosis	2 (7)
Osteoarthritis	9 (29)
Psoriatic arthritis	1 (3)
Rheumatoid arthritis	10 (32)
Spondylosis (cervical/lumbar) Psychodiagnosis	6 (19)
GAD ^1^	16 (52)
MDD ^2^	3 (10)
GAD ^1^ + MDD ^2^	12 (38)
Pain intensity (M, SD)	8.12 (1.78)
Fatigue	8.09 (2)
Anxiety	6.54 (2.89)
Depression	6.35 (2.52)

Abbreviations: ^1.^ Generalized Anxiety Disorder; ^2.^ Major Depressive Disorder.

**Table 4:** Correlations between baseline measures.

Dimension	Pain	Fatigue	Anxiety	Depression	Experiential Avoidance	Cognitive Fusion
Hurt Connect	0.40 *	−0.07	0.07	−0.04	0.24	0.31
Experience Range Emotions	−0.31	0.06	−0.17	−0.17	−0.39 *	−0.36 *
Thinking Got In Way	0.22	0.09	0.42 *	0.13	0.33	0.30
Important Challenge	−0.08	0.49 **	0.01	0.23	−0.10	−0.10
Stuck Unable Change	−0.08	−0.37 *	−0.08	−0.21	−0.11	−0.10
Thinking Helped Life	−0.29	0.18	−0.13	0.04	−0.41 *	−0.35 *
Struggle Connect Moments	−0.23	0.27	−0.20	−0.06	−0.35 *	−0.30
Connect To People	−0.12	0.41 *	−0.10	−0.06	−0.29	−0.33
Personal Import	−0.09	0.41 *	−0.09	0.13	−0.22	−0.16
Hurt Health	0.17	−0.01	0.11	0.11	0.45 **	0.60 **
Pain	-	0.34	0.40 *	0.24	0.29	0.43 *
Fatigue		-	0.23	0.41 *	0.07	0.02
State Anxiety			-	0.14	0.37 *	0.28
State Depression				-	0.16	0.18
Experiential Avoidance					-	0.60 **

* *p* < 0.05; ** *p* < 0.01 (2-tailed).

**Table 5:** Summary of primary outcomes at T1 after the single-session treatment.

Outcome Measure T1	Mean (SD)		t (30)	*p* Value	Effect Size	
	Pre-Test	Post-Test T1			(Cohen’s *d*)	Hedges’ g
Cognitive Fusion	28.19 (17.77)	17.77 (6.58)	9.96	0.001	1.78 (1.21; 2.35)	1.74 (1.18; 2.29)
Experiential avoidance	28.06 (6.86)	19.84 (6.38)	7.79	0.001	1.4 (0.89; 1.89)	1.36 (0.87; 1.84)
Able To Change Behavior (PBAT_1)	64.84 (22.19)	81.61 (20.67)	−4.37	0.001	−0.78 (−1.18; −0.37)	−0.76 (−1.15; −0.36)
Helped Health (PBAT_6)	60.00 (24.76)	79.68 (19.40)	−4.32	0.001	−0.77 (−1.17; −0.36)	−0.75 (−1.14; −0.36)
Complying (PBAT_9)	61.61 (27.21)	41.94 (22.12)	3.86	0.001	0.69 (0.29; 1.08)	0.67 (0.28; 1.05)
Stuck To Working Strategies (PBAT_10)	45.81 (29.18)	28.39 (25.04)	3.27	0.003	0.58 (0.2; 0.96)	0.57 (0.19; 0.94)
Hurt Health (PBAT_17)	45.16 (25.41)	25.48 (22.03)	4.05	0.001	0.72 (0.32; 1.12)	0.71 (0.31; 1.09)

**Table 6:** The qualitative analysis of main improvements for cognitive fusion, experiential avoidance and behavioral processes at T1.

Outcome Measure T1	N (%)	
	Mild Improvement (≥25%)	Moderate Improvement (≥50%)
Cognitive Fusion	16 (52)	9 (29)
Experiential avoidance	15 (49)	5 (16)
Able To Change Behavior (PBAT_1)	8 (26)	4 (13)
Helped Health (PBAT_6)	9 (29)	8 (26)
Complying (PBAT_9)	5 (16)	8 (26)
Stuck To Working Strategies (PBAT_10)	6 (19)	12 (39)
Hurt Health (PBAT_17)	3 (10)	13 (42)

**Table 7:** Summary of outcomes at the T2 one-month assessment.

Outcome Measure	Mean (SD)		Before Imputation Analysis				After Imputation Analysis			
	Pre-Test	Post-Test			Effect Size				Effect Size	
			t (26)	*p* Value	(Cohen’s *d*)	Hedges’ g	t (30)	*p* Value	(Cohen’s *d*)	Hedges’ g
Pain	8.26 (1.65)	5.70 (2.52)	6.43	0.001	1.23 (0.72; 1.73)	1.2 (0.7; 1.68)	6.21	0.001	1.11 (0.65; 1.56)	1.08 (0.64; 1.52)
Fatigue	7.96 (2.06)	7.33 (2.03)	1.55	0.174	-	-	1.97	0.057	-	-
Anxiety	7.11 (2.57)	4.96 (2.68)	4.45	0.001	0.85 (0.4; 1.29)	0.83 (0.39; 1.25)	3.08	0.004	0.55 (0.17; 0.93)	0.54 (0.16; 0.9)
Depression	6.26 (2.56)	4.15 (2.69)	3.43	0.002	0.66 (0.23; 1.04)	0.64 (0.23; 1.04)	3.96	0.001	0.72 (0.31; 1.12)	0.7 (0.31; 1.08)

**Table 8:** Time effect estimates for dependent variables.

	Estimate	95% CI	SE
Cognitive Fusion	10.41	8.28; 12.55	1.04
Experiential avoidance	8.22	6.07; 10.38	1.05
Able To Change Behavior (PBAT_1)	−16.77	−24.6; −8.94	3.83
Helped Health (PBAT_6)	−19.67	−28.98; −10.37	4.55
Complying (PBAT_9)	19.67	9.28; 30.07	5.09
Stuck To Working Strategies (PBAT_10)	17.41	6.54; 28.29	5.32
Hurt Health (PBAT_17)	19.67	9.76; 29.58	4.85
Pain	2.38	1.6; 3.17	0.38
Anxiety	1.58	0.53; 2.62	0.51
Depression	2.25	1.05; 3.36	0.56

## Data Availability

The data presented in this study are available on request from the corresponding author (cosmin.popa@umfst.ro).
